# The Oxidative Stress and Chronic Inflammatory Process in Chagas Disease: Role of Exosomes and Contributing Genetic Factors

**DOI:** 10.1155/2021/4993452

**Published:** 2021-12-23

**Authors:** Edio Maldonado, Diego A. Rojas, Fabiola Urbina, Aldo Solari

**Affiliations:** ^1^Programa Biología Celular y Molecular, Instituto de Ciencias Biomédicas, Facultad de Medicina, Universidad de Chile, Santiago, Chile; ^2^Instituto de Ciencias Biomédicas, Facultad de Ciencias de la Salud, Universidad Autónoma de Chile, Santiago, Chile

## Abstract

Chagas disease is a neglected tropical disease caused by the flagellated protozoa *Trypanosoma cruzi* that affects several million people mainly in Latin American countries. Chagas disease has two phases, which are acute and chronic, both separated by an indeterminate time period in which the infected individual is relatively asymptomatic. The acute phase extends for 40-60 days with atypical and mild symptoms; however, about 30% of the infected patients will develop a symptomatic chronic phase, which is characterized by either cardiac, digestive, neurological, or endocrine problems. Cardiomyopathy is the most important and severe result of Chagas disease, which leads to left ventricular systolic dysfunction, heart failure, and sudden cardiac death. Most deaths are due to heart failure (70%) and sudden death (30%) resulting from cardiomyopathy. During the chronic phase, *T. cruzi*-infected macrophages respond with the production of proinflammatory cytokines and production of superoxide and nitric oxide by the NADPH oxidase 2 (NOX2) and inducible nitric oxide synthase (iNOS) enzymes, respectively. During the chronic phase, myocardial changes are produced as a result of chronic inflammation, oxidative stress, fibrosis, and cell death. The cellular inflammatory response is mainly the result of activation of the NF-*κ*B-dependent pathway, which activates gene expression of inflammatory cytokines, leading to progressive tissue damage. The persisting production of reactive oxygen species (ROS) is the result of mitochondrial dysfunction in the cardiomyocytes. In this review, we will discuss inflammation and oxidative damage which is produced in the heart during the chronic phase of Chagas disease and recent evidence on the role of macrophages and the production of proinflammatory cytokines during the acute phase and the origin of macrophages/monocytes during the chronic phase of Chagas disease. We will also discuss the contributing factors and mechanisms leading to the chronic inflammation of the cardiac tissue during the chronic phase of the disease as well as the innate and adaptive host immune response. The contribution of genetic factors to the progression of the chronic inflammatory cardiomyopathy of chronic Chagas disease is also discussed. The secreted extracellular vesicles (exosomes) produced for both *T. cruzi* and infected host cells can play key roles in the host immune response, and those roles are described. Lastly, we describe potential treatments to attenuate the chronic inflammation of the cardiac tissue, designed to improve heart function in chagasic patients.

## 1. Introduction

Chagas disease or American trypanosomiasis is a tropical vector-borne disease, and its etiologic agent is the protozoan parasite *Trypanosoma cruzi*. It was discovered in 1909 by the Brazilian researcher Carlos Chagas and is endemic to South and Central America, including Mexico, however is not endemic in the Caribbean islands and Puerto Rico [1]. A Pan American Health Organization (PAHO) estimate indicates that 8-12 million people are infected in endemic areas and another 100 million people are at risk of infection. In the past two decades, the disease has been spread to other areas, namely, the United States, Europe, and Western Pacific areas, due to human immigration [2–7]; therefore, Chagas disease is a worldwide concern. In the United States, it is estimated that there are over 300.000 infected people by *T. cruzi*; meanwhile, in Europe, it is estimated to be around 80.000 infected people [1, 3, 7]. In endemic areas, Bolivia has the highest incidence and prevalence rates; however, Brazil, Argentina, and Colombia have a higher number of infected people, due to their larger population and also due to the fact that a large population lives in rural areas, where the vector triatomine bugs are present. The distribution of the triatomine bugs has been changing, and now, Chagas disease is spreading to periurban and urban regions of those countries [1, 3].

Currently, vaccines are not available to prevent infection, although many South American countries have applied programs of interrupting vectorial transmission, which has led to significantly reduced new infections [5]. Also, public health guidelines, such as education of risky populations and serological screening of blood donors, have reduced the transmission of Chagas disease [1, 3, 6]. Despite all those efforts, it is estimated that approximately 60.000 new infections are produced each year and around 15.000 people can die each year from Chagas disease [1, 3, 7].

The disease is transmitted by several species of bloodsucking triatomine insects (invertebrate host), and one of the most common vectors in South America is *Triatoma infestans* [3, 5]. The insects get *T. cruzi* as the infective trypomastigote by sucking the blood of wild or domestic mammals, which act as reservoirs of the parasites; then, the trypomastigotes differentiate to the replicative epimastigotes and multiply in the insect midgut and finally are excreted as metacyclic trypomastigotes in the feces/urine during or immediately after a blood meal [1, 3]. The trypomastigotes enter the vertebrate host bloodstream through the bite wound or can enter through mucosal membranes and proliferate inside the host cells as amastigotes. Chagas disease has two phases, and each of them presents different clinical syndromes. The initial acute phase can last approximately 6-8 weeks and often is asymptomatic or unrecognized due to the mild symptoms; however, around 5% of the infected people can present a severe acute phase, mainly children under 5 years old, elderly, and immunosuppressed patients [3]. This severe form of the disease can cause fulminant myocarditis, which can produce patient death due to heart failure [2]. During the acute phase, there is a high detectable parasitemia level. This acute phase usually resolves spontaneously, and the patients remain infected if they are left untreated. This indeterminate form of Chagas disease has a good prognosis, and most of the infected people never develop symptoms of cardiac or digestive failure, despite being seropositive for *T. cruzi* [2, 3]. However, a decade or up to three decades after the initial infection, around 30-40% of the chronically infected people can progress to a chronic phase and develop organ damage, mainly cardiomyopathy and megaviscera (megaesophagus, megacolon, or both of them) [2, 3]. The most serious and frequent complication of Chagas disease is chronic cardiomyopathy, which is characterized by fibrosis and chronic inflammation resulting in heart failure [2].

Heart tissue inflammation is the hallmark of chronic Chagas disease; however, the pathogenesis of chronic chagasic cardiomyopathy is not completely understood yet. Until recently, the consequences of the chronic disease are considered to be autoimmune, but later, it became clear that autoimmune reaction is a consequence of parasite persistence. However, other factors must be considered to explain chagasic cardiomyopathy. In this article, we will review recent findings on the pathogenesis of the disease, role of reactive oxygen species (ROS) in keeping on the chronic heart inflammation of Chagas disease, the host immune response, the role of exosomes in Chagas disease, and new perspectives on patient treatments.

### 1.1. Pathophysiology of Chagas Disease

As stated, the most frequent complications of Chagas disease are syndromes affecting cardiac, digestive, and neurological systems of the patients. The late manifestations of the chronic phase of the disease are disturbances in the heart, such as cardiomyopathy and cardiac arrhythmias, esophageal or colon motility, and neurological damage. These late manifestations typically present as cardiac arrhythmias, cardiomyopathy, or disturbances in esophageal and colon motility involving hypertrophy and dilatation of the organ. The underlying anatomical abnormalities in Chagas disease patients with cardiac damage are an enlarged heart with parasympathetic denervation and reduced ganglion cell numbers in the myenteric plexuses [8]. This reduction in ganglion cell numbers is assumed to occur in the acute phase of the infection, as it has been demonstrated through comparative studies [9–12]. The cardiac system is the most compromised and affects about 30% of chagasic patients, while megaesophagus and megacolon are less frequent (5-20%) [2, 3]. A mixed form of chronic Chagas disease (cardiac and digestive) affects only 5-10% of the chagasic patients [3]. Death from chronic Chagas disease is the result of congestive heart failure that follows the development of myocardial dysfunction, caused by a chronic inflammatory response that might progress to fibrosis and cell death; however, intestinal denervation also occurs as a result of *T. cruzi* infection [3, 13, 14]. At least three factors are involved in the pathogenesis of Chagas disease, namely, (i) the induction of autoimmune response, (ii) the cellular inflammatory response, and (iii) oxidative stress generated as a consequence of *T. cruzi* infection [15–19].

The pathophysiology of the acute and chronic phases of Chagas disease is quite different. The acute phase presents high parasitemia and tissue parasitism, but with a preference for the heart, esophagus, colon, and central nervous system. The presence of *T. cruzi* elicits a strong immune/inflammatory response against the parasite. In the heart, diffuse myocarditis is present together with myocyte necrosis, cell infiltration (mononuclear and polymorphonuclear), and interstitial edema [2, 3, 19]. On the other hand, chronic Chagas disease presents persistent inflammation, which results in diffuse myocytolysis and appearance of reparative fibrosis [2, 3, 19]. Subsequently, a ventricular dysfunction appears together with reduced myocardial function, heart remodeling, and neurohumoral activation [19]. Together, the effect of all those factors results in a cycle of disease progression, which can lead to heart failure. Cellular injuries and the strong immune/inflammatory response are the main sources of reactive oxygen species (ROS), and the mitochondria are the major source of ROS during the chronic phase of Chagas disease [19, 20]. Oxidative stress produced by mitochondrial ROS (mtROS) contributes to tissue damage and sustains oxidative stress in the myocardium [19, 20]. In the following section, the new evidence on the role of oxidative stress on myocardium damage will be described.

## 2. Inflammation and Oxidative Damage in Chagas Disease

**Figure 1:**
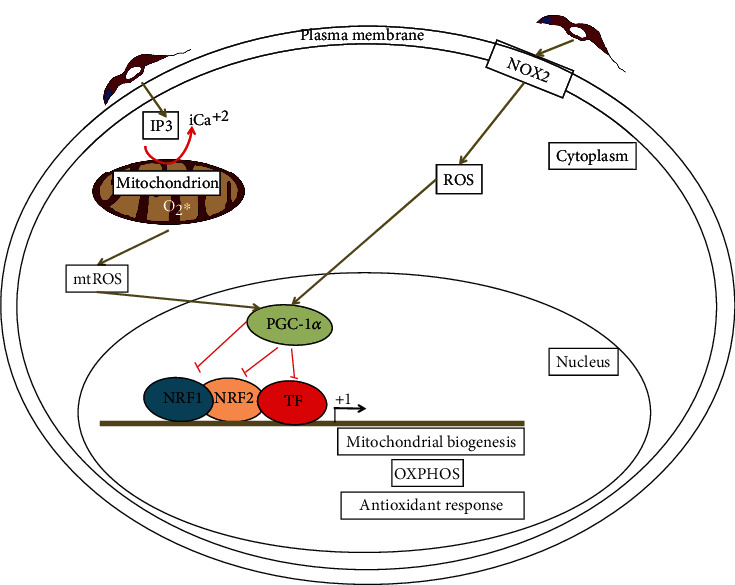
A mechanism to explain the mitochondrial dysfunction in Chagas disease. NOX2 is activated in *T. cruzi*-infected monocytes and macrophages, which can produce ROS. On the other hand, *T. cruzi* infection can induce an intracellular calcium flux (iCa^2+^), which in turn causes mitochondrial membrane permeability, respiratory complex malfunction, and electron leakage from the electron transport chain to oxygen, which results in increased mtROS production. Most likely, the iCa^2+^ is associated with changes in the inositol 1,4,5 trisphosphate (IP3) signaling pathway, which can cause Ca^2+^ release from the sarcoplasmic reticulum and the nuclear envelope. Both NOX2-produced ROS and mtROS can inhibit the activity of the transcriptional coactivator PGC-1*α*, a coactivator of NRF1, NRF2, and other transcription factors (TF) involved in mitochondrial biogenesis, OXPHOS, mtDNA replication, and transcription, altering the redox homeostasis of the mitochondria. Additionally, ROS could directly inhibit the activity of NRF1 and NRF2, leading to mitochondrial dysfunction as well. +1 represents the transcription start site of the genes.

Chronic inflammation and oxidative stress are hallmarks of chronic cardiomyopathy in Chagas disease. When a host is infected by *T. cruzi*, there is an attempt to control the infection by elevating the levels of ROS and NO, which leads to oxidative stress at both the acute and chronic phases of Chagas disease [20]. There are two main ROS sources relevant to this disease which are NOX2 and mitochondria (mtROS), and a proposed mechanism of mitochondrial dysfunction caused by ROS is described in Figure 1. Several studies have demonstrated that NOX2 locates at the plasma membrane of peritoneal mouse macrophages upon interaction with the parasite [20, 21], and it has been also demonstrated that *T. cruzi*-activated macrophages can produce high levels of ROS and NO both in *in vitro* and *in vivo* models [20, 21]. Also, it has been shown that splenocytes from infected mice and *in vitro* cultured macrophages can respond to *T. cruzi* infection by activating NOX2, which results in increased levels of ROS [20]. The heart is a target organ for *T. cruzi* infection, and infiltrating neutrophils and macrophages are the main sources of NOX2- and myeloperoxidase-dependent ROS in the acute phase of the Chagas disease, although it has been also observed that mitochondria can produce ROS from infected cardiomyocytes [20]. Both ROS and NO can react and form peroxynitrite, which is able to kill *T. cruzi* inside the macrophages; however, peroxynitrite can be harmful to the host cell as well. ROS can also act as key regulators of parasite control by means of regulating cytokine responses and also splenic inflammatory cell proliferation during the infection [20]. However, recent evidence indicates that ROS can provide a positive signal for *T. cruzi* cell proliferation [22].

Signaling lymphocyte activation molecule family 1 (SLAMF1 or CD150) of cell-surface receptors is broadly expressed in the hematopoietic system [23]. SLAMF1 can be considered a phenotypic marker of activated T cells, dendritic cells, B cells, and monocytes. It has been shown that it is a key player in innate and adaptive immunity and serves as a bacterial sensor and as a receptor for *Morbilliviruses* [23]. It has been reported that interaction between OmpC/F+ *E. coli* and SLAMF1 is required for macrophage phagocytosis and phagosome localization where it can enhance PI3P production, NOX2 activation, and superoxide production [24]. Superoxide has antimicrobial activity and also is involved in regulating cell motility and phagocytosis; therefore, the phagocytosis of Gram- bacteria can be compromised in the absence of superoxide [25]. In the context of *T. cruzi* infection, SLAMF1 controls the susceptibility of infection by the virulent Y strain, since *Slamf1*−/− mice, lacking the SLAMF1 receptor, are resistant to a lethal *T. cruzi* Y strain challenge [26]. Moreover, Slamf1-defIcient myeloid cells are impaired in their ability to replicate the parasite, and they display an altered pattern of cytokine production. Recent studies by Poveda et al. [27] have shown that the interaction of SLAMF1 with *T. cruzi* is strain-dependent and affects NOX2 expression and ROS production, since five out of six strains tested showed a decrease in parasite load in *Slamf1*−/− infected macrophages compared to wild-type macrophages. Also, NOX2 expression and ROS production were increased in *Slamf1*−/− infected macrophages when compared to wild-type macrophages [27]. Those results indicate that SLAMF1 increases parasite infection and controls ROS production through NOX2.

When ROS are produced in excess or for long and sustained periods, they exert toxic effects that damage cells and tissues, since ROS can oxidize lipids, proteins, and DNA [20, 28]. These effects produce cell membrane damage, which leads to membrane integrity loss and membrane protein function defects. The main damage on molecules produced by ROS is lipid peroxidation, a direct attack on Thr, Pro, Lys, and Arg residues of proteins that can derivatize the polypeptides and lead to the formation of protein carbonyls [20, 28–31]. DNA can be oxidized and damaged to produce 8-oxo-deoxyguanosine (8-oxo-G) lesions, which might exceed the capacity of the cellular DNA repair mechanisms, leading to mutations and transcription mistakes [31]. The ROS in chagasic hearts is produced by dysfunctional mitochondria, since it has demonstrated a decline in complex I (CI) and complex III (CIII) activities, which are associated with excessive electron leakage to produce superoxide and sustained ROS production in the myocardium of chagasic mice [31–33].

ROS play a pivotal role as signaling intermediates to link the innate and adaptive immune responses by generating proinflammatory cytokine production (TNF-*α*, IL-1*β*, and IFN-*γ*) from dendritic cells and macrophages, which are components of the innate immune system [20]. Studies in which the NOX2/ROS axis was inhibited in primary and cultured macrophages have demonstrated that NOX2/ROS is a key regulator of cytokine production in response to *T. cruzi* infection [34]. Subsequent studies using splenocytes from infected mice in vitro stimulated with *T. cruzi* antigens have confirmed those observations and demonstrated that inhibition of NOX2 by apocynin or by using ROS scavengers greatly blocked the phagocyte cell proliferation and production of inflammatory mediators such as IL-1, IL-6, TNF-*α*, and IFN-*γ* [34–36]. Most importantly, a genetic deficiency of a subunit of NOX2 (p47phox) resulted in an augmented susceptibility to *T. cruzi* infection in those mice lacking the p47phox gene, suggesting that the redox state plays a key role in immune system activation and control of the *T. cruzi* infection [20, 29, 37].

## 3. Diseased Heart and Mitochondrial Biogenesis

The heart consumes enormous amounts of energy, and it is highly dependent on the mitochondria for the energy required for its contractile and other metabolic activities. In the cardiomyocytes, mitochondria represent around 30% of the total cell volume and provide 90% of the cellular ATP energy through the oxidative phosphorylation (OXPHOS) pathway [38]. Creatine kinase reaction serves as the heart primary energy reserve, and the creatine phosphate shuttle system delivers the high-energy phosphate groups from their production site in the mitochondria to the myofibrils to regenerate the ATP consumed during heart contraction [38]. This ATP flux is reduced in heart failure.

Heart development occurs mainly during perinatal and postnatal periods, when a series of maturation events start including mitophagy, fusion, and fission of the mitochondria [38]. This process allows the redistribution and dense packing of mature mitochondria along the myofibrils and also allows fatty acid oxidation (FAO), which are the predominant fuel substrate for the adult heart. The biogenic machinery can coordinate the nuclear and mitochondrial genomes during development and also in response to the source of fuel substrate utilization or energy demands [38]. Mitochondrial biogenesis, maturation, and function are controlled at the transcriptional level by a master regulator, which is the transcriptional coactivator named peroxisome proliferator-activated receptor *γ* (PPAR*γ*) coactivator-1*α* (PGC-1*α*) [39]. This coactivator is strongly activated by conditions causing energy limitation, such as cold, exercise, and fasting, and is particularly highly expressed in organs demanding large energy consumption, such as the heart [40, 41]. PGC-1*α* binds to and activates PPARs, which are key regulators of genes involved in FAO, such as PPARs, hepatocyte nuclear factor 4, and SIRT1 [40, 41]. It also functions as a transcriptional coactivator for the nuclear respiratory factors 1 and 2 (NRF1 and NRF2) and triggers the expression of genes involved in mitochondrial biogenesis, OXPHOS, transcription, and replication of the mitochondrial genome [40, 41]. PGC-1*α* also serves as a transcriptional coactivator for estrogen-related receptors (ERRs), which can act as amplifiers for PGC-1*α* activation of PPARs and NRFs [36, 42]. PGC-1*α* is also a coactivator for the transcription factor nuclear factor-erythroid-derived 2-like 2 (NFE2L2, also known as NRF2, however is different from nuclear respiratory factor 2), which plays a key role in inducible expression of several detoxification and cytoprotective genes involved in response to oxidative and electrophilic stresses. SIRT1 is the main regulator of PGC-1*α* and activates PGC-1*α* through a NAD^+^-dependent deacetylation at specific lysine residues of PGC-1*α*, and since NAD^+^ levels are regulated by AMPK during fasting, the activation of PGC-1*α* through deacetylation potentially creates a link between energy status, redox status, and mitochondrial function [42, 43].

Heart failure and cardiac hypertrophy in nonchagasic individuals are characterized by an increase of gene expression of many genes normally expressed in the fetal heart along with a decreased expression of those genes usually expressed in the adult heart [44]. This organ can meet its energy requirements from the oxidation of fatty acids, glucose, lactate, and other oxidizable substrates. However, the heart can function better when it uses both fatty acids and glucose simultaneously [45]. During heart failure, the heart switches from fatty acids to glucose utilization with a downregulation of the enzymes involved in FAO, which implies a reversion to the fetal energy substrate preference [45, 46]. The expression of genes involved in FAO, which are PGC-1*α*-coactivated, is downregulated during cardiac hypertrophy, and this is even prior to a clear cardiac dysfunction [47]. Cardiac hypertrophy is associated with reduced myocardial fatty acid utilization, and this observation indicates that mitochondria are remodeled to a phenotype with a reduced capacity to perform FAO during cardiac hypertrophy development and heart failure [44]. The loss of PGC-1*α* accelerates cardiac dysfunction following pressure overload stress [48]. Moreover, germline deletion of PGC-1*α* evokes perinatal lethal heart failure, which is caused by a lack of cardiac mitochondrial biogenesis [44, 49]. Interestingly, a subset of mitochondria in adult PGC-1*α*-knockout mice displays ultrastructural cristae abnormalities, which resembles those observed in the Bart syndrome, a congenital disease caused by altered phospholipid biosynthesis [44, 50]. PGC-1*α* as a transcriptional coactivator can interact directly with members of the nuclear receptor superfamily of transcriptional activators via LXXL recognition motifs, recruiting molecules that mediate chromatin remodeling by histone acetylation and interactions with the mediator complex to recruit the RNA polymerase II transcription machinery [51]. Effector transcriptional factors within this cascade include members of the PPAR, ERR, NRF1, and NRF2 and factors involved in the transcription and replication of the mitochondrial genome.

In the context of Chagas disease, *T. cruzi*-infected cardiomyocytes and human chagasic hearts show increased mRNA levels, but a decreased nuclear localization of PGC-1*α*-coactivated transcription factors such as NRF1, NRF2, and NFE2L2 [52]. However, there was an enhancement of gene expression of genes involved in PPAR*γ*-regulated FAO, NRF1- and NRF2-activated mtDNA replication, and transcription machinery gene such as the mitochondrial transcription factor A (TFAM) [52]. Mitochondrial DNA content and mtDNA replication decrease by 65% and 83%, respectively. ROS, oxidative stress, and mtDNA oxidation are significantly increased, and the NFE2L2-antioxidant gene expression was severely compromised in infected cardiomyocytes and in human chagasic hearts [52]. The impairment of mitochondrial biogenesis by the decrease of the mtDNA content caused by the defect in the mtDNA replication results in a significant loss of mitochondrial-encoded proteins of the OXPHOS pathway [52]. It is most likely that the oxidated mtDNA is not a template to support replication and gene expression. The mtDNA replication defects increase ROS generation and a functional incapacity of NFE2L2 to activate genes of the antioxidant response leading to oxidative stress in the cardiomyocytes [52]. However, the PGC-1*α*-coactivated NRF1 and NRF2 transcriptional activities in the expression of genes involved in mtDNA replication and transcription were not compromised; rather, the defects in cardiomyocyte mitochondrial function can be attributed to a loss of function of NFE2L2 [52].

## 4. Mitochondrial Dysfunction and ROS Production in Chagas Disease

Mitochondrial ROS can be produced from infected cardiomyocytes as a consequence of mitochondrial dysfunction and contributes to increased oxidative stress in the heart of chagasic individuals. Usually, in the mitochondria occurs a low, but constant, ROS production [31]. The main sites for electron leakage are CI and CIII, which leads to increased ROS generation in the mitochondria [53, 54]. Wen and colleagues [14, 33] have demonstrated that in chagasic mice, there is a decline in CI and CIII activities in the myocardium, and this was associated with excessive electron leakage to O_2_ and increased superoxide formation, resulting in a sustained ROS production in chagasic mice. In those mice, there was a significant decline in the content of mtDNA, and mitochondria-encoded transcripts were also diminished; therefore, quantitative deficiencies in the respiratory chain activity are produced in chagasic mice [33]. As a consequence of the mitochondrial dysfunction, OXPHOS-mediated ATP synthesis capacity of the myocyte mitochondria is reduced. This is extremely important since the heart is highly dependent on mitochondria for energy requirement for its contractile activity and the mitochondria provide more than 90% of the cellular ATP energy through OXPHOS. Importantly, the mitochondria are targets of several insults, which include inflammatory mediators produced during Chagas disease. Further studies by the same group [33] have identified that CIII is the main source of ROS production in the infected myocardium of chagasic mice. Electron leakage is produced as a consequence of defects in CIII proximal to the Qo site in cardiac mitochondria of *T. cruzi*-infected mice [33]. The excessive electron leakage can be reduced by the treatment of infected mice with the antioxidant phenyl-a-tert-butyl-nitrone (PBN), which is a spin-trapping antioxidant [33]. PBN can improve the respiratory chain function by preventing the excessive mtROS production in myocyte mitochondria and reduce electron leakage. Mitochondrial defects at CIII have been also observed in the heart of chronic Chagas patients [55], indicating that mitochondria are the main source of ROS production and oxidative stress during the chronic phase of Chagas disease. Moreover, the ROS-induced oxidative adducts can conduce to proinflammatory macrophage activation, which in turn can damage the mitochondria, leading to an increase in mtROS production [55].

**Figure 2:**
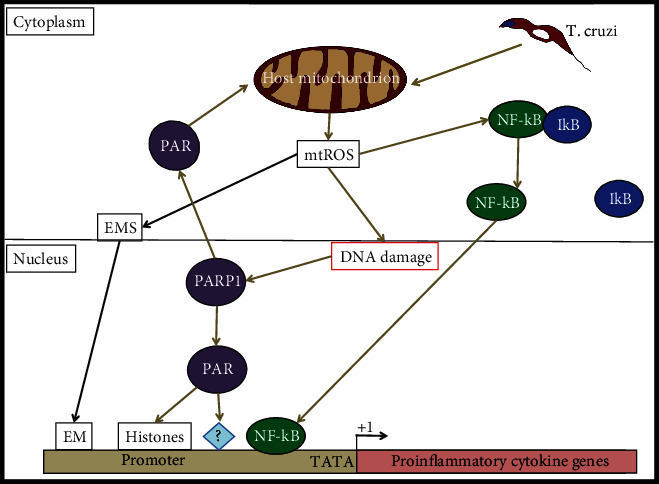
The persisting ROS signal activates proinflammatory gene expression. *T. cruzi*-infected cardiomyocytes are able to produce mtROS, which induces the dissociation of NF-*κ*B transcription factor from I*κ*B in the cytoplasm and translocate to the nucleus to bind target sequences in the gene promoter of proinflammatory cytokine genes, which leads to the high production of proinflammatory cytokines, which in turn can produce tissue damage. Mitochondrial ROS can produce DNA damage and activate PARP1 to produce PAR, which signals transcriptional coactivators to induce proinflammatory cytokine gene expression and modify histones at the gene promoter regions. Alternatively, ROS could activate an epigenetic modification system (EMS) that might be able to produce an epigenetic modification (EM) at the promoter regions of proinflammatory cytokine genes to keep them in an active transcribing state, even though in the absence of *T. cruzi*. +1 represents the transcription start site of the genes.

Mitochondrial ROS has a profound impact on cytokine gene expression in cardiac myocytes infected with *T. cruzi*. Garg and colleagues have demonstrated that mtROS augmented the nuclear translocation of the v-rel avian reticuloendotheliosis viral oncogene homolog A (RelA or p65), therefore activating the NF-*κ*B-dependent gene expression of inflammatory cytokines such as TNF-*α*, IL-1*β*, and IFN-*γ* [56]. Also, mtROS can cause 8-oxo-G lesions and DNA fragmentation, which can signal poly-ADP ribose polymerase 1 (PARP1) to get modified by poly-ADP-ribose (PAR) together with other proteins in infected cardiomyocytes [20, 57–59]. PARP1 can signal other proteins, such as histones, by PARylation; however, the hyperactivation might have negative effects, such as catalytic activation of inflammatory proteins, enhanced cytokine gene expression activation, NAD^+^ depletion, and cell death [20, 57–59]. Importantly, inhibition of PARP1 expression by RNAi, chemical inhibition by PJ34, or removing mtROS by an antioxidant was beneficial to block mtROS formation and DNA damage [20, 56, 59]. Interestingly, PARP1 can also regulate cytokine gene expression by a different mechanism, which is the PAR modification of RelA (p65)-interacting nuclear proteins and the assembly of a NF-*κ*B transcription-dependent complex [20]. All those studies indicate that the ROS-PARP1-RelA signaling pathway can contribute to the proinflammatory cytokine gene expression in *T. cruzi*-infected cardiomyocytes. Those results are summarized in Figure 2.

The mechanisms underlying the progression to the chronic phase of Chagas disease in humans are still under study. It is noteworthy to state that even in the absence of *T. cruzi*, the individual can develop a chronic disease as evidenced by the BENEFIT trial [60, 61]. The mechanisms that trigger the progression to the chronic phase of Chagas disease are not completely known yet. During the chronic phase of the disease, there is a high production of mtROS by the cardiomyocytes as a consequence of changes in the mitochondrial membrane potential (ΔΨ) due to lipid peroxidation and/or formation of protein carbonyls and a decline in the activities of the respiratory complexes, especially CIII [20, 33]. The persisting ROS release leads to mitochondrial dysfunction, inflammation, and heart tissue damage. Cellular components such as lipids, proteins, and DNA are damaged which can lead to proinflammatory macrophage activation causing cellular injury [55]. The high ROS levels produce exhaustion of the antioxidant defense of the organism, which is mainly the superoxide dismutase (MnSOD), glutathione peroxidase, catalase, and glutathione [37, 61–64]. Therefore, the use of antioxidant compounds, stimulation of mitochondrial biogenesis, and enhancement of antioxidant defenses or ROS scavengers could have benefits in the treatment of chronic Chagas disease. Recently, it has been found that TNF-*α*+monocytes/macrophages are associated with the inflammatory process in chronic Chagas disease. There is an increase in the maturation of bone marrow hematopoietic stem cell-derived monocytes of proinflammatory and anti-inflammatory phenotypes in chronic *T. cruzi*-infected mice [65]. On the other hand, yolk sac-derived monocytes/macrophages of the CD11b+ F4/80+ phenotype were augmented in sinusoidal compartments of chagasic mice [65]. The splenic monocytes/macrophages of that phenotype displayed augmented mRNA, protein, and surface expression of proinflammatory markers, such as CD80+/CD64+, which are associated with cytokine response (TNF-*α*, IL-6), and those cells are also detected in the myocardium of chagasic mice [65]. Taken all together, those results indicate that proinflammatory yolk sac-derived monocytes/macrophages trigger the splenic and myocardium inflammatory response.

## 5. Innate Immune Response in Chagas Disease

ROS are important mediators that participate in the immune response by triggering the production of cytokines by immune cells. This section will analyze the main aspects of the innate immune response mounted by the host during *T. cruzi* infection.

When invading a vertebrate host, *T. cruzi* must face the first defense line with the innate immunity composed of phagocytes, especially macrophages, neutrophils, and dendritic cells [66]. Those cells have two main features that are key to their functions, which are (i) recognition of the pathogen-associated molecular patterns (PAMPs) and the damage-associated molecular patterns (DAMPs) by membrane receptors, such as the Toll-like receptors (TLR). This enables the cells to recognize and phagocyte foreign microorganisms and cellular debris to destroy those invading microorganisms. (ii) Secretion of cytokines which promote inflammation and activate other cells involved in defense at the site of infection. Pattern recognition receptors (PRR) can recognize several shared motifs in different microorganisms, such as the classical lipopolysaccharides present in the cell wall of bacteria, which are able to activate the expression of genes involved in inflammation and antimicrobial responses. *T. cruzi* is also recognized by the nucleotide-binding oligomerization domain-like receptors or Nod-like receptors (NLRs), which are intracellular sensors of PAMPs that can enter the cells via phagocytosis or pores and also of DAMPs, which are associated with cell stress [67]. On the other hand, TLR is a family composed of 10 different members in humans [68]. Some of them are at the cell surface and others into the cell. TLR2 and TLR4 are surface receptors, while TLR7 and TLR9 are located at the endosome. For example, glycosyl phosphatidylinositol- (GPI-) anchors, which are derived from *T. cruzi* mucin-like glycoproteins (GPI-mucins), are ligands of TLR2 and 6 [69], while glycol-inositol-phospholipids (GIPLs) are ligands of TLR4 [70]. Meanwhile, DNA (rich in unmethylated CpG motifs) and RNA are potent activators of TLR9 and 7, respectively [71, 72].

All those recognition signals regulate the expression of proinflammatory cytokines from macrophages, such as IL-1, IL-12, TNF-*α*, and IL-10 [73]. Those proinflammatory cytokines, together with IFN-*γ*, which is produced by natural killer (NK) and T cells upon *T. cruzi* infection, are able to prime macrophages, which leads to the induction of the iNOS system that can produce high amounts of NO for 24 hours. The NO can diffuse to the phagosome where it can react with O_2_ to produce ONOO-, a powerful oxidant able to kill *T. cruzi* [73]. Dendritic cells, like macrophages and neutrophils, also become activated in the presence of PAMPs and DAMPs to produce costimulatory molecules and cytokines that are necessary, together with the antigen itself, to activate T cells [74]. The profile of the produced cytokines by the dendritic cells will depend on the nature of the invading pathogen and can direct the differentiation of naive T cells towards different functional roles. Experiments with human dendritic cells have shown that their functions are affected by factors secreted by *T. cruzi* that can induce tolerance (no response) by inhibiting TNF-*α* and IL-12 production, even though the parasite was internalized into the cytoplasm of the dendritic cell [75]. Furthermore, a decrease in molecules of classes I and II of the major histocompatibility complex (MHC) and CD-40 coreceptor expression was observed [75]. Those decreases are induced by *T. cruzi* soluble factors, interfering with the antigen presentation ability of dendritic cells. Despite the fact that the parasite is internalized by the dendritic cell, there are different degrees of infectivity, which is dependent upon the *T. cruzi* strain, however is not dependent on the *T. cruzi* discrete typing units (DTUs) and neither dependent on the biological characteristics of the dendritic cells [76].

Also, NK cells play a key role in innate immunity against intracellular pathogens and in contrast to T and B lymphocytes do not require clonal expansion or differentiation to carry out their functions [66]. NK cells can signal T cell differentiation towards an inflammatory pathway and also can secrete IFN-*γ* to activate macrophages when viable *T. cruzi* infecting cells are present [66]. For example, it has been shown that a peak of IFN-*γ* is produced soon after infection by *T. cruzi*, in a process which requires adherent cells in the thymus and viable parasites, however is independent of T cells [77]. This IFN-*γ* peak might be key in the control of the infection during the acute phase of Chagas disease, as the depletion of NK cells from those mice causes an abrogation in IFN-*γ* production and an increase in IL-10 levels, which probably leads to tolerance of the parasite and allows infection progression [77]. In addition, NK cells can directly eliminate extracellular parasites by forming intercellular contacts with the pathogen cell, which results in the loss of motility and damage in the cell membrane of the parasite [66, 78, 79]. This killing activity of the NK cells depends on activation by IL-12 and results in the exocytosis of cytotoxic granules, which can damage the cell membrane of the parasite [66, 78, 79]. It is believed that NK cell primary function is the direct killing of the parasite, more than the elimination of *T. cruzi*-infected cells [66, 79]. It is noteworthy to mention that expression of genes related to NK cell's function is upregulated in PCR-positive asymptomatic patients with Chagas disease, while they are downregulated in those patients with severe cardiomyopathy [80].

**Figure 3:**
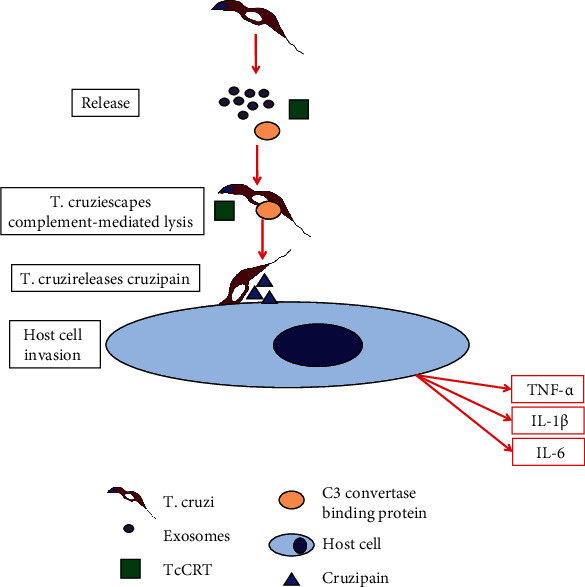
Cellular and molecular mechanisms performed by exosomes during *T. cruzi* infection. *T. cruzi* releases exosomes during infection, which contain C3 convertase binding protein and most likely contains TcCRT as well. C3 binds to the *T. cruzi* surface, but its cleavage is inhibited by C3 convertase binding protein, therefore inhibiting the complement pathway and escaping complement-mediated lysis. On the other hand, TcCRT binds C1q and mannose-binding proteins and ficolins, inhibiting the classical and lectin complement pathways. The parasite also releases cruzipain, which helps *T. cruzi* to invade the host cell. Infected host cells can release cytokines such as TNF-*α*, IL-1*β*, and IL-6. Symbols associated with each molecule or cells are indicated in the box.

The complement cascade system is another component of innate immunity. It consists of several plasmatic proteins, which are able to opsonize pathogens, recruit phagocytes to the infection site, and also directly destroy the invading pathogen [66]. It functions as a cascade of proteolytic enzymes, in which a precursor (zymogen) is activated turning it up in an active proteolytic enzyme able to cleave and activate the following component [66, 79]. The complement system works by amplifying the signal generated by the presence of the pathogen to allow the clearance of the invading pathogen. It can be activated by three different vias, which are the classical, the alternative, and the lectin pathway, but all of them converge in the component C3 convertase and its subsequent split in C3a and C3b [81]. Neutrophils and macrophages have receptors for C3b and promote phagocytosis of the pathogen, whereas C3a acts as a proinflammatory factor. C3b also acts on C5 to produce C5a, a potent proinflammatory factor, and also is able to generate pores in the pathogen cellular membrane [81]. However, despite the power of the complement system to control initial infections, *T. cruzi* has evolved a myriad of molecules to escape or subvert the complement system, by either inactivation or blocking the activation of its components. For example, calreticulin (TcCRT) is a *T. cruzi* endoplasmic reticulum protein, which is translocated to the surface upon infection, and it has been demonstrated that it can bind to several molecular pattern sensors, such as C1q, mannose binding lectin, and L-ficolin [66, 79, 82], thereby affecting the first step on the classical and lectin complement system pathway. Several other examples of complement system inactivation by *T. cruzi* molecules can be found in Reference [66, 79].  Figure 3 shows that a C3 convertase binding protein is contained in *T. cruzi* exosomes, and it is able to block its activity. We believe that TcCRT can also be contained into the *T. cruzi* released exosomes.

## 6. Adaptive Immune Response in Chagas Disease

B lymphocytes play a fundamental role in adaptive humoral immune response since they are able to produce and secrete antibodies. Moreover, B cells can secrete cytokines and are involved in antigen presentation to other immune cells, thereby acting as a nexus between innate and adaptive immune responses [66, 79]. B cells and their antibodies were one of the first components studied in the context of Chagas disease; however, several aspects of their roles are not well understood yet. The focus of interest during the early days of the field was the specificity of the elicited antibodies generated upon infection [66, 79]. Today, the immune epitope database (IEDB) has more than 90 *T. cruzi* molecules entries and several others marked as “other *T. cruzi* proteins” displaying more than 2.000 different epitopes, which can be bound by antibodies from human patients and animal models. Some of those antibodies are lytic since they can bind to the parasite and activate the complement system for parasite lysis [83]. Therefore, those epitopes bound by neutralizing antibodies might be good candidates to generate vaccines against the parasite. Those antibodies are mostly directed against surface antigens on *T. cruzi*, and a description of several of the antigenic molecules can be found in [66, 79]. The advances of the high throughput technologies and bioinformatics have considerably expanded the possibility to analyze the complete repertory of antibodies after infection, and moreover, a database of antigen binding regions of the antibodies could be built to serve as tools for the study of the humoral response in Chagas disease [84].

The importance of the humoral immune response to control the *T. cruzi* infection has been studied in the mouse infection model, and it has been shown that mutant mice, unable to produce antibodies, cannot control the *T. cruzi* growth and die during the acute phase of the disease [85]. Those results indicate the importance of the B cells to control the infection at the beginning of Chagas disease. However, antibodies produced against *T. cruzi* seem to not effectively control and completely eliminate the parasite, providing the opportunity for the parasite to establish a permanent infection [66, 79]. This inefficient humoral immune response could be due to three main factors: (i) antigenic variability on the surface antigens, which are encoded by multigene families and the high diversity of molecules expressed at the same time, delays the activation of specific B cell clones that impedes the production and maturation of high-affinity specific antibodies with lytic and neutralizing activities; (ii) reduced number of immature B cells in the bone marrow, most likely as a consequence of increased apoptosis in the bone marrow, due to *T. cruzi* infection. By affecting the bone marrow, *T. cruzi* compromises the entire humoral response, causing the decrease of mature B cells in the periphery; and (iii) nonspecific polyclonal B cell activation, since some parasite antigens have been demonstrated that can cause a nonspecific T cell-independent activation of B cells in mouse models of infection. That activation of the B cells can cause splenomegaly and hypergammaglobulinemia with production of antibodies that are unspecific for *T. cruzi* [86]. This could in part explain the pathogenesis of chronic Chagas disease, since those antibodies could react with host antigens, and evidence of molecular mimicry has been found between host and parasite antigens [66, 79, 87].

The B cell-deficient murine model has been used to study, during the acute phase of Chagas disease, the role of B cells in the function of T cells. In those mice, a deficient expansion of CD4+ and CD8+ T cells was observed, especially within the memory T cell subset, and also a decrease in TH1, TH17, and regulatory T cell populations [88, 89]. This indicates that B cells are important for the establishment of memory T cells, which are essential to control *T. cruzi* infection. Moreover, in the absence of mature B cells, the number of IFN-*γ* CD4+ T cells decreases, while the number of TNF+ CD4+ T cells increases with a concomitant augment of the cytokine levels in plasma [66, 88, 89]. Additionally, the frequency of CD4+ T cells expressing inhibitory receptors is lower than that in wild-type infected mice, indicating that the regulation of those cell populations has been hampered. All of those defects can contribute to the deleterious and proinflammatory state of those mutant mice [88, 89].

T lymphocytes or T cells play a key role in adaptive cellular immune response. A T cell response is initiated by signals, which are produced by a recognition of peptide-MHC complexes presented on the surface of antigen-presenting cells (APC), by the T cell receptors [66, 79, 90]. The consequence of this activation is the generation of a large number of effector and pathogen-specific activated T cells from a relatively small population of naive T (Tn) cells with different specificities and roles. Tn cell activation triggers their clonal expansion, along with changes in the molecular expression, especially membrane-anchored receptors, and cytokine production, which can enable the T cell effector capabilities [66, 79, 90]. In the context of an acute infection, such as *T. cruzi* infection, the T cell response can be divided into three phases: (i) priming and expansion, (ii) resolution and contraction, and (iii) memory acquisition [66, 79, 90]. During the first phase, the Tn cells proliferate and differentiate in effector T (TE) cells, which increase the expression of several molecules, such as surface receptors and chemokine receptors, which favor activation and migration to lymphoid organs to be retained there [66, 79, 90]. Those changes upon activation promote a rapid amplification of that cell population for a specific response, generation of effector and memory cell populations, enhancement of the APC, and a response within nonpathological levels. There are two types of TE; those monofunctional produce only one cytokine, whereas those polyfunctional simultaneously produce more than one cytokine. In the context of infection and vaccination, the production of polyfunctional cells is considered a good correlate of protection [91, 92]. After the elimination of the antigen source (pathogen), although this is not exactly the case with Chagas disease, the clonal contraction step takes place. Most of the activated TE die for apoptosis, and the immune system goes back to its homeostatic state [93]. However, the process of activation of Tn cells not only produces specific TE cells but also gives rise to memory T cells that can remain after the contraction phase is finished [92]. Those can self-renew by proliferation and can be perpetuated in the long term, being remarkably efficient to acquire effector functions when a new challenge with the antigen occurs [66, 79, 93]. As stated earlier, *T. cruzi* infection can bypass the innate and humoral immunity, and the disease can progress to a chronic state. In chronic Chagas patients, it has been demonstrated that there are an increased number of circulating activated T cells, which can secrete pro- and anti-inflammatory cytokines [94]. However, there is a hampered T cell proliferative response in Chagas disease patients, when cells are exposed to a strong nonspecific mitogen, and there is also a decreased expression of T cell receptors involved in activation [95, 96]. Moreover, T cell activation from noninfected individuals is inhibited by *T. cruzi* antigens [95, 96]. T cell response must be important to maintain a low parasitemia during the chronic phase of Chagas disease, and whether impaired by coinfections (e.g., VIH) could lead to a rapid clinical onset of the disease. On the contrary, whether this response is exacerbated could lead to a pathological state, which must be controlled to impede disease progression and tissue damage. During the host adaptive immune response and Chagas disease progression, the exosomes produced by the parasite play a pivotal role, which is described below.

## 7. Genetic Variants Associated with Mitochondrial Dysfunction and Inflammation in Cardiomyopathies in Chagas Disease

Chronic Chagas disease cardiomyopathy is an inflammatory cardiomyopathy which is prevalent in Latin America and is present in 30% of the six million individuals infected with *T. cruzi*; however, 70% of the infected individuals remain asymptomatic or free of heart disease. As mentioned earlier, IFN-*γ* and mitochondrial dysfunction play a main pathogenic role in this disease. Very little is known about the genetic contribution to the progression of chronic inflammatory cardiomyopathy in chronic Chagas disease and the factors that increase cardiomyocyte susceptibility to inflammatory damage. A recent and interesting exome sequencing analysis was performed to study nuclear families containing multiple cases with chronic Chagas inflammatory cardiomyopathy and chronic-infected asymptomatic siblings and in unrelated but infected asymptomatic individuals [97]. Heterozygous pathogenic variants are linked to chronic Chagas disease in all tested families on twenty-two distinct genes; twenty were mitochondrial or inflammation-related, with most of them involved in proinflammatory cytokine production [97]. It has been suggested by the authors that chronic Chagas cardiomyopathy-linked genetic variants can increase mitochondrial susceptibility to IFN-*γ*-induced damage to the myocardium, which leads to the observed phenotype of chronic inflammatory cardiomyopathy [97]. This was only found in individuals that were both seropositive and carriers of the heterozygous pathogenic variants which developed chronic Chagas cardiomyopathy, but not those seropositive patients carrying the wild-type sequences, neither seronegative siblings carrying the pathogenic variant. The finding that only a single family carried a variant in a gene previously associated with familiar cardiomyopathy indicates that the genetic landscape and pathogenesis of chronic Chagas disease cardiomyopathy is distinct from that of familial cardiomyopathy [97]. Among the nine mitochondrial genes showing chronic Chagas disease cardiomyopathy-specific pathogenic variants, eight are involved in mitochondrial ATP synthesis (biogenesis, translation, FAO, and OXPHOS). Previous studies have shown that patients carrying mutations or animals genetically deficient in six mitochondrial genes (described in 97) can develop cardiac phenotypes [98–101]. Notably, up to 30% of mitochondriopathy patients develop cardiomyopathy, heart conduction defects, ventricular arrhythmia or sudden cardiac death, and autonomic nervous system imbalance [102], while up to 15% develop gastrointestinal motility disorders including achalasia/megaesophagus and megacolon [103]. There is a striking similarity between the clinical presentation and the proportion of cardiac and digestive disorders in mitochondriopathies and the clinical spectrum of chronic Chagas disease, which suggests that the pathogenesis of Chagas disease might be the consequence of mitochondrial dysfunction as well. Chronic Chagas disease cardiomyopathy displays signs of reduced mitochondrial respiratory chain activity [32, 104], energy production [105], decreased rRNA [106], rDNA [107], and decreased ATP production [104]. There is a loss of ΔΨ in cardiomyocytes with a concomitant increase in ROS production [108]. In exome sequencing analysis, another eleven pathogenic variants were located in ten inflammation-associated genes. Eight out of these genes are involved in proinflammatory cytokine production via activation of NF-*κ*B and MAP kinase pathways, which leads to an increase in proinflammatory cytokine production, which could lead to further mitochondrial dysfunction in cardiomyocytes. This mitochondrial damage can be further enhanced by IFN-*γ*. Dysfunctional mitochondria have an increased ROS production, which can produce oxidative stress, further damaging the mitochondria. Interestingly, patients carrying heterozygous gene variants had a normal childhood and reported not to have a debilitating disease before developing chronic Chagas cardiomyopathy as adults, and it is inferred that the genetic variants alone by themselves were not able to induce childhood-onset mitochondriopathy [97]. Finally, the authors support the notion of a two-hit mechanism where IFN-*γ* and proinflammatory cytokines induced by chronic *T. cruzi* infection trigger mitochondrial dysfunction and clinical disease in carriers of heterozygous mitochondrial gene variants [97].

## 8. Role of Exosomes in Chagas Disease

The exosomes are membrane-bound extracellular vesicles secreted into the extracellular space by endothelial cells (ECs) and several other cells such as immunocytes, platelets, and smooth muscle cells [109–111]. They play key roles in cell-to-cell signaling, and they are present in almost all biological fluids [112, 113]. There is a continuously extracellular exchange of exosomes, containing bioactive molecules, in between organelles to promote communication during homeostasis and diseased states of the organism [113]. Several studies have suggested the participation of exosomes in cellular communication associated with physiological and pathological states due to their ability to modify the recipient cell phenotype [114, 115]. The process of exosome generation begins with the internalization of the cellular membrane through endocytosis to form an endosome [116]. Afterwards, an invagination occurs on the endosomal membrane to end up with the maturation of multivesicular bodies (MVBs). These MVBs can either be degraded by internal lysosomes or transported to the cell membrane to undertake transcytosis or fusion and release of the contents into the extracellular space as exosomes [116]. Exosomes can then target cells through specific receptors binding to activate cell-to-cell signaling pathways such as horizontal gene transfer, inflammation, antigen presentation, tumor progression, and mediation of the immune response during pathogenic states [117, 118]. The contents of the exosomes consist of several metabolites, proteins, lipids, RNA, and DNA, which can be interchanged in between exosomes and their target cells. Exosomes have been isolated from protozoa, bacteria, viruses, and fungi; however, each exosome has different compositions [119]. As in the case of protozoan parasites, those can modulate host cell responses by producing exosomes with virulence factors and effector molecules and this way can modulate host gene expression, immune response, and factors to favor parasite growth, survival, and pathogenesis [120]. On the other hand, exosomes released during *T. cruzi* infection might also make possible a host immune response [121].

As in the context of Chagas disease, the parasite and host cell exosomes play a fundamental role in the pathogenesis of Chagas disease. *T. cruzi* infection induces blood cells to release exosomes by a Ca^2+^-mediated mechanism [122]. The released exosomes are essential to host-parasite interactions, intercellular communications, and increased parasite survival. As an example, exosomes can protect extracellular trypomastigotes from the action of the complement through the binding of C3 convertase on the parasite surface, inhibiting C3 cleavage [123, 124]. Also, exosomes released by *T. cruzi* promote cell invasion and parasite survival by modulating the innate immune system and producing several virulence factors including glycoprotein 85 (gp85), trans-sialidase, phosphatase, and other soluble proteins [124–126]. Therefore, the released parasite and cell host exosomes are able to play a key role during parasite invasion of the host innate immune system, parasite survival, and infection establishment in Chagas disease [127].  Figure 3 shows that *T. cruzi* released exosomes can contain molecules to escape complement-mediated lysis.

**Figure 4:**
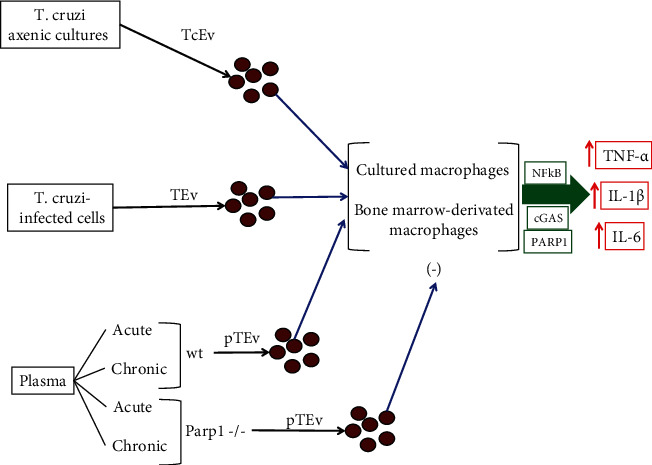
*T. cruzi* extracellular vesicles can activate macrophages to release cytokines. Extracellular vesicles from *T. cruzi* axenic cultures (TcEv), infected cells (TEv), or cultured cells of both acutely or chronically infected mice (pTEv) can stimulate both cultured and bone marrow-derived macrophages to secrete TNF-*α*, IL-1*β*, and IL-6. However, plasma-derived TEv from mutant Parp1-/- mice cannot induce cytokine production in macrophages, and it was determined that induction of cytokine production is through the PARP1-cGAS-NF-*κ*B axis.

Under pathological states, the stimulus that activates exosome formation can regulate their selective arrangement of constituents and composition, thereby regulating the biological information that can transfer. It has been recently demonstrated that exosomes produced by *T. cruzi* trypomastigotes can be fused to host cell membranes and promote exosome release from THP-1 macrophages [128, 129]. Also, it has been found that human peripheral blood mononuclear cells incubated with *T. cruzi* can secrete exosomes, and those exosomes elicit a proinflammatory gene expression response in human THP-1 macrophages. Similarly, a proinflammatory cytokine response was observed in THP-1 macrophages when incubated with exosomes isolated from peripheral blood of Chagas disease patients [130]. Thus, those findings indicate that exposure to *T. cruzi* can influence exosome release, and those have a deep impact on the surrounding infected or injured tissue. The mechanisms of exosome-dependent macrophage activation by *T. cruzi* are not well understood yet. In a recent study, Choudhuri and colleagues [131] have studied the role of exosomes in producing the macrophage response in progressive Chagas disease. It was found that cultured and bone marrow-derived macrophages can respond to exosomes produced from axenic parasite cultures, *T. cruzi*-induced exosomes produced by infected cells, and exosomes derived from plasma of acutely or chronically infected mice [131]. All of those can induce a profound increase in expression and release of cytokines such as TNF-*α*, IL-6, and IL-1*β*. Exosomes produced by immune cells (macrophages) and nonimmune cells (muscle) were proinflammatory. However, exosomes derived from plasma of mutant PARP1-/- significantly reduced the exosome-induced transcriptional and translational activation of proinflammatory macrophage response. Interestingly, oxidized DNA contained into the exosomes was necessary for PARP1-dependent proinflammatory response, and the studies suggested that DNA-sensing immune receptor cyclic GMP-AMP synthase (cGAS), a PRR, synergized with PARP1 to signal and activate the NF-*κ*B pathway [131]. Inhibition of both PARP1 and cGAS resulted in more than 80% inhibition of the exosome-induced NF-*κ*B pathway activity. In chagasic mice, a severe inflammatory infiltrate was found, which was associated with an intense increase in CD11b+CD68+TNF-*α*+ macrophages [131]. In contrast, mutant PARP1-/- chagasic mice showed a low-to-moderate tissue inflammation and more than 80% diminution of myocardial infiltration by TNF-*α*+ macrophages, and there was no change in immunoregulatory IL-10 macrophages [131]. A schematic representation and the results obtained from those experiments are shown in Figure 4.

Although macrophages are the principal immune cells that can exert trypanocidal effects, since they produce ROS and NO, it has been found that nonimmune cells, such as skeletal muscle cells and cardiomyocytes, can produce high levels of ROS in response to *T. cruzi* infection [77, 132]. ROS can exert cytotoxic effects by oxidizing cellular components including proteins, lipids, and DNA and cannot discriminate between parasite or host cellular components. Indeed, it has been shown that 8-oxo-G, which is a marker for oxidative DNA damage, is augmented in *T. cruzi*-infected cardiomyocytes and in the myocardium of chagasic mice and in chronic Chagas disease patients [59]. Those damaged encapsulated DNA molecules serve as a stimulus for proinflammatory activation of macrophages [131]. The authors proposed that genomic damaged DNA from both host and parasite, which is into the exosomes, provides the primary stimulus in engaging DNA sensing innate immune receptors and macrophage activation in the context of Chagas disease progression [131]. However, it is unknown whether the damaged DNA are random sequences, or it has some sequence preference. Thus, there is an axis PARP1-cGAS-NF-*κ*B, which activates the proinflammatory macrophage activation by exosomes released during *T. cruzi* infection and during the progression of Chagas disease.

## 9. Omics Studies of *T. cruzi*-Host Cell Interactions

Chagas disease is a clinical consequence of *T. cruzi* infection, and cardiomyopathy is the most severe consequence of the chronic phase of the disease, which cannot be reversed only by reducing the parasite load in the patient. The interaction of *T. cruzi* with host cells is able to trigger several molecular signaling cascades, and the response will be dependent on the cell type, *T. cruzi* strain, and also experimental variables. The global responses of host cells can be effectively studied by -omics technologies, especially transcriptomics and proteomics to assess the gene expression state of *T.cruzi*-infected cells. Even though total gene expression levels can provide useful information, it should be remembered that many important signaling pathways are mediated by posttranscriptional modifications of a particular polypeptide, with minimal variation in protein levels. In particular, posttranscriptional modifications such as phosphorylation, acetylation, and glycosylation are important modifications to regulate the activity of proteins. Therefore, total gene expression levels should be complemented with proteomic approaches that could be combined with phosphoproteomic studies to dissect precisely the signaling pathways involved in chronic *T. cruzi* infection.

It is well known that different *T. cruzi* strains present biological differences including virulence, pathogenicity, and infectivity. To understand the variability, the different strains are classified into six main groups, which are known as discrete typing units (DTUs, from TcI to TcVI). However, there exists a high genetic and biological difference even between strains at the intra-DTU level that can impact the host response to *T. cruzi* infection. Therefore, we would like to highlight that the specific strain impacts *T. cruzi* host cell interactions, and studies with one strain could be not comparable with another different strain, even though they belong to the same DTU.

Transcriptomic studies focused on the changes in the respiratory chain and OXPHOS of host cells, in response to *T. cruzi* infection, have found an upregulation of energy-related metabolism pathways in human primary cardiomyocytes at early times using the Dm28c strain (TcI) [133]. However, when the Tulahuen strain (TcVI) is used, those changes were not observed. In the context of mouse primary cardiomyocytes and at early time postinfection with the Dm28c strain, there were no significant changes in the pathways related to energy metabolism [134], although in other similar studies, but using the Brazil strain (TcI), a downregulation of the electron transport activity was observed, though this study was done on late time postinfection [135]. All these studies indicate that the transcriptomic results are dependent on the *T. cruzi* strain, origin of the infected cell, and the experimental conditions used to perform the study. In the context of in vivo transcriptomics, it has been shown that mouse hearts infected with the Sylvio strain (TcI), Brazil strain (TcI) and Y strain (TcII), Col1.7G2 strain (TcI), and JG strain (TcII) showed a decrease in the expression of genes related to energy metabolism (reviewed in [136] and references therein). Regarding chronic Chagas patients, the cardiac gene expression profile showed an increase in genes related to energy metabolism (reviewed in [136] and references therein). Again, those studies suggest that in vivo transcriptomic results depend on the origin of the infected cell.

Studies that evaluated the changes of both the transcriptional level and the functional response of the components related to cellular respiration have been performed by several groups (reviewed in [135]). In human primary cardiomyocytes with the Dm28c strain, an increase in respiration and mitochondrial biogenesis was found [133], and also, Shah-Simpson et al. [137] found that *T. cruzi*-infected human dermal fibroblasts had increased respiration and mitochondrial biogenesis. Human-infected macrophages with Sylvio strain had also an increase in respiration [138]. Opposite results have been found using murine-infected cells. A decrease in respiration has been found in murine-infected cardiomyocytes with the CLBr strain (TcVI) at similar analyzed times as it was done with human cells [139]. Also, a decrease in the respiration complexes CI and CIII was found in infected murine cardiomyocytes with Sylvio strain [99]. Those studies indicate that the results are related to the specific origin of the cells (mouse or human). In the context of *in vivo* infections, when evaluating the protein expression of different respiratory complexes, a decreased expression in the complexes CI-CV in cardiac mitochondria of Sylvio-infected mice was found [32]. It has also been found to have a decreased mitochondrial function in the heart, skeletal muscle, colon, and stomach of acute infected mice and in hearts and stomach of chronic *T. cruzi*-infected mice [62]. There are few studies analyzing the cellular respiration in tissues or cells derived from chronic Chagas patients. As an example, in an analysis of hearts from a group of five patients, Teixeira et al. [140] found that there was a small decrease in the expression levels of the alpha subunit of the ATP synthase enzyme (CV). In a similar work done by Wan et al. [52], analyzing the heart of eight chronic Chagas disease patients has shown a decrease in the mitochondrial Cyt B complex protein (CIII) and ND1 protein (CI). Considering that there are several cell types present in the heart (fibroblasts, endothelial cells, and cardiomyocytes) and additional infiltrating macrophages and T cells in the heart of chagasic patients, it might be important to carry out studies in a larger number of chagasic heart samples with isolated cardiomyocytes as long as possible.

Multiplexed proteomic studies in the heart of a Chagas disease mouse model, at the chronic phase of the disease, have revealed an increase in the immune response and strong repression in the expression of several mitochondrial proteins [141]. Concomitantly, the phosphoproteomic analysis showed abundance in phosphosites in plasma membrane and cytoskeletal proteins. The analysis of kinase activity evidenced an activation of the JNK/p38 MAP kinases and also activation of the DYRK2 and AMPKA2 kinases. However, the casein kinase family was inhibited in the host response to *T. cruzi* infection. As it was expected, there was an increase of the IFN-*γ*-mediated signaling pathways [89] and repression of the mitochondrial function [21, 29, 142].

Significantly, new players that might be important for disease progression were identified in the study [141], such as Immunity-Related GTPase M (IRGM) 1 and 2 and also the immune-associated guanylate binding proteins (GBPs). Additionally, to changes in total protein abundance, it was uncovered a vast signaling network of plasma membranes and intermediate filament proteins with altered phosphorylation status after *T. cruzi* infection. Those include proteins such as Striated Muscle Enriched Protein Kinase (SPEG), Tensin 1, Sorbin and SH3 domain-containing protein (SORBS) 1/2, BCL2 Associated Athanogene (BAG3), and proteins from the myosin family. In those studies, the authors highlighted signaling pathways that can be further studied and validated for their contribution to the disease progression and also could be potential drug targets.

Finally, caution should be taken to interpret and extrapolate results from the murine model to humans, since as mentioned above, an increase in the respiration of *T. cruzi*-infected human cells is found; however, a decrease in infected cells of murine origin is usually found. Libisch and colleagues [136] have proposed that mice have a lower ability to maintain adequate cellular homeostasis against different types of stress, such as oxidative stress by ROS, since mice have a metabolic rate per gram of body weight approximately seven times higher compared with humans [136]. ROS is normally generated [143] but is highly increased in *T. cruzi*-infected cardiomyocytes (mtROS) and macrophages [99, 144]. Perhaps, this is the main reason to explain why cells of human or murine origin do not present the same response against *T. cruzi* infection, at least respecting pathways related to energy metabolism. It is also likely that differences in the immune systems between humans and mice could have an impact on mitochondrial respiration, since a relationship has been established between the immune system and energy metabolism [136, 145].

## 10. Chagas Disease Treatments

**Figure 5:**
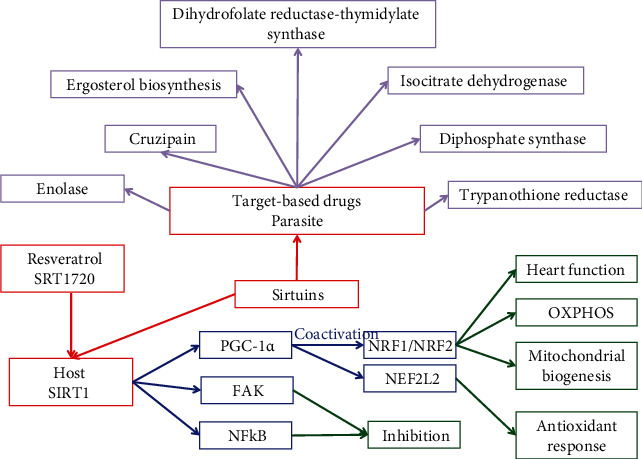
Target-based drugs against *T. cruzi* and SIRT1 therapy. Drugs are being developed against specific *T. cruzi* pathways, and they are shown in the figure. Both the parasite and the mammalian host possess sirtuins; therefore, target-based drugs have been tested against the parasite sirtuins, and antioxidant therapies have been developed to improve the activity of mammalian SIRT1, either by using resveratrol antioxidant or the SRT1720 agonist, which is able to activate SIRT1, which in turn can activate PGC-1*α* to promote mitochondrial biogenesis and OXPHOS function and improve heart function through the transcription factors NRF1 and NRF2. Also, PGC-1*α* is a coactivator for NFE2L2, which can activate the antioxidant system. On the other hand, activated SIRT1 can inhibit the activity of transcription factor NF-*κ*B, which leads to a decrease of proinflammatory cytokine production, and also can inhibit the activity of FAK.

The main therapy for Chagas disease treatment is benznidazole (BZN); however, this drug is highly toxic for patient treatments at the chronic phase and is more effective at the acute phase of the disease [146]. New therapies propose the reduction of BZN doses for chemotherapeutic treatment combined with other drugs to slow the progress of the disease. One example is the combination of the trypanolytic BZN combined with simvastatin to prevent endothelial cell activation induced by *T. cruzi* infection [147]. In this study, the anti-inflammatory effect of simvastatin is mediated by the inhibition of NF-*κ*B. Another study in mice with the antioxidant resveratrol in chronic *T. cruzi* infection improved cardiac function, activating the AMPK pathway without altering the heart inflammatory infiltrates or vascularization [148]. Similar results were observed in a study using carvedilol alone or combined with vitamins E and C to treat patients with chronic chagasic cardiomyopathy. The combined use resulted more efficiently in reducing the oxidative damage, but both treatments were unable to control the inflammatory process, as evaluated by the increase of the inflammatory markers, such as adenosine deaminase and myeloperoxidase [149]. However, when an anti-inflammatory therapy using ibuprofen was compared with an antioxidant therapy using vitamins E and C in *T. cruzi*-infected mice, the first resulted more efficient in the attenuation of oxidative stress and cardiac damage, without altering cardiac parasitism [150]. The use of the anti-inflammatory administration of aspirin does not alter the parasitological course of *T. cruzi* infection in mice, however reduced cardiac inflammatory infiltrates and thromboxane levels [151]. Another type of drug being developed to treat Chagas disease is the so-called target-based drugs to interfere with specific pathways of *T. cruzi* [152]. There are several of those; however, the most important are drugs that can target the ergosterol biosynthesis pathway, trypanothione reductase, cruzipain, enolase, ribose-5-phosphate isomerase, sterol 14-*α*-demethylase, pteridine reductase, farnesyl diphosphate synthase, isocitrate dehydrogenase 2, dihydrofolate reductase-thymidylate synthase, and the sirtuins [152]. A schematic view of those approaches can be found in Figure 5. On the other hand, new drug candidates with trypanocidal activities have been recently developed, and details can be found in Reference [153]. Sirtuins are deacetylase enzymes of eukaryotic origin, which can regulate several cellular processes, and they have been described as potential targets for Chagas disease treatment [154]. Both *T. cruzi* and the mammalian host have sirtuins. Mammalian SIRT1 is one of the main sirtuins and one of the most studied. SIRT1 therapy is one of the most promising potential new therapies to treat Chagas disease at the chronic phase and deserves a brief analysis. The antioxidant resveratrol, which activates SIRT1, which in turn can deacetylase PGC-1*α* to activate it and increase the mitochondrial number, stimulates the gene expression of genes involved in oxidative phosphorylation (OXPHOS) function and activates the antioxidant defenses through the NRF factors [155]. SRT1720 is a small selective molecule that can activate SIRT1 by binding to the SIRT1-substrate complex. This small SIRT1 activator is 1,000-fold more potent than resveratrol and can improve OXPHOS function and attenuate aging-related cardiomyocyte dysfunction [156–158]. Its use during the chronic phase of Chagas disease can restore the heart left ventricular function, and PGC-1*α* was in an active state in chagasic mice, although mitochondrial biogenesis was not improved [155]. SRT1720 treatment of *T. cruzi*-infected mice can decrease splenic expansion and infiltration of proinflammatory monocytes/macrophages in the chagasic mice; however, the capabilities of those cells to respond to *T. cruzi* stimulus are not altered. SRT1720 decreases the *T. cruzi*-induced augment of expression and/or phosphorylation activity of focal adhesion kinase (FAK) and downstream target transcription factors such as Pu.1, c-Myb, and Runx1, involved in macrophage proliferation and migration and Notch1 involved in functional cell activation [55, 65]. Studies using cultured macrophages confirmed that SRT1720 can control the *T. cruzi*-induced FAK-dependent expression of those transcription factors and demonstrated that SRT1720 and a FAK specific inhibitor [55, 65] can inhibit the NF-*κ*B transcriptional activity and in turn inflammatory cytokine gene expression in *T. cruzi*-infected macrophages. Taken altogether, those results indicate that STR1720 can reprogram the *T. cruzi*-induced FAK-dependent transcription factors needed for proliferation and proinflammatory activation in Chagas disease.

SRT1720 treatment can reduce ROS, nitrosative stress, and inflammatory response in the chagasic myocardium through the inhibition of the NF-*κ*B transcriptional pathway, with the subsequent inhibition of the production of proinflammatory cytokines [155]. The main mechanism by which SIRT1 inhibits NF-*κ*B is by a direct deacetylation of p65/RelA preventing the release and nuclear translocation of NF-*κ*B, although other mechanisms can also contribute to the SIRT1 activity [155, 157]. The authors also concluded that *T. cruzi*-induced inhibition of the SIRT1/PGC-1*α* regulatory axis is not a key mechanism in mitochondrial biogenesis defects observed in Chagas disease [155, 157]. Combined treatment of BZN and SRT1720 could be beneficial during the acute phase of Chagas disease to avoid disease progression and also during the chronic phase of the disease to improve heart function. In this context, we suggest that treatment with activators or agonists of SIRT3 might be another option for the treatment of Chagas disease.

## 11. Concluding Remarks and Future Directions

It seems clear that chronic cardiomyopathy in Chagas disease is the result of several factors such as the parasite itself, host adaptive immune response, oxidative stress, inflammatory stress, and mitochondrial dysfunction. However, the megaviscera and neurological disorders are another manifestation of chronic Chagas disease and have not been studied as well as chronic cardiomyopathy, and it is an area that also deserves attention. Mitochondrial dysfunction in cardiomyocytes leads to the production of mtROS, which can induce DNA damage and signal PARP1 to produce PAR, which can help to activate the expression of proinflammatory cytokine genes. Also, mtROS can activate the NF-*κ*B transcription factor, which acts as a transcriptional activator on proinflammatory cytokine genes increasing its expression, which leads to inflammatory stress with sustained ROS production and therefore produces oxidative damage of the cardiac cellular components during the chronic Chagas disease. At the acute phase of the disease, the host innate and the adaptive immune can control the parasitic invasion; however, in around 30% of the patients, the infection is able to progress, and it produces a chronic phase of the disease that can be manifested decades later of the initial infection. The parasite can escape the innate and adaptive immune response by a series of mechanisms such as blocking the complement pathway by producing molecules that can inhibit the complement components or by producing a nonspecific immune response. Secreted extracellular vesicles (exosomes) from both the parasite itself and from the infected host cells play a key role in the immune response. The parasite secretes exosomes containing molecules that can inhibit the complement components and exosomes carrying molecules which can induce cytokine production and a host immune response.

Future studies should be focused to investigate the nature of the mitochondrial dysfunction which produces the mtROS and the persisting ROS signal that produces the inflammatory and oxidative stress states in the cardiac tissue, which is responsible for the cardiac tissue damage. It is likely that epigenetic mechanisms triggered by mtROS could be able to maintain the proinflammatory cytokine gene expression, either by modifying their promoters to keep them on or by activating the gene expression or activity of the NF-*κ*B transcription factor or the transcriptional coactivators necessary for the expression of the proinflammatory cytokine genes. The exosome molecular contents should also be identified, and their contribution to the host immune response has to be studied. Since there is not an available vaccine yet, drugs to treat chronic Chagas disease should be developed and those able to target specific metabolic pathways in *T. cruzi* are especially promising, and also, those drugs able to improve heart function in chagasic patients will be extremely valuable.

## Abbreviations

APC: Antigen-presenting cell
BZN: Benznidazole
CI, CII, CIII, CIV: Respiratory chain complexes
cGAS: GMP-AMP synthase
DAMPs: Damage-associated molecular patterns
*Δ*MIT: Membrane potential
DTU: Discrete typing unit
ERR: Estrogen-related receptor
FAK: Focal adhesion kinase
FAO: Fatty acid oxidation
GPI: Glycosyl phosphatidylinositol
IFN: Interferon
IL: Interleukin
iNOS: Inducible nitric oxide synthase
MHC: Major histocompatibility complex
MnSOD: Manganese-dependent superoxide dismutase
mtROS: Mitochondrial ROS
MVBs: Multivesicular bodies
NAD^+^: Nicotinamide adenine dinucleotide
NFE2L2: Nuclear factor erythroid 2-related factor 2
NF-*κ*B: Nuclear factor kappa-light-chain-enhancer of activated B cells
NLR: Nod-like receptor
NOX2: NADPH oxidase 2
NO: Nitric oxide
NRF: Nuclear respiratory factor
OXPHOS: Oxidative phosphorylation
PAMPs: Pathogen-associated molecular patterns
PAR: Poly-ADP-ribose
PARP1: Poly-ADP-ribose polymerase 1
PGC1*α*: Peroxisome proliferator-activated receptor *γ* coactivator 1*α*
PPAR: Peroxisome proliferator-activated receptor *γ*
PRR: Pattern recognition receptors
RelA: v-rel avian reticuloendotheliosis viral oncogene homolog A
ROS: Reactive oxygen species
SIRT1: Sirtuin 1
SLAMF1: Signaling lymphocytic activation molecule 1
TE: Effector T cell
TcCRT: Calreticulin
TLR: Toll-like receptors
Tn: Naive T cell
TNF: Tumor necrosis factor.
